# Impact of the medical fitness model on long term health outcomes in older adults

**DOI:** 10.1186/s12877-024-05208-6

**Published:** 2024-08-20

**Authors:** Ranveer Brar, Alan Katz, Thomas Ferguson, Reid Whitlock, Michelle Di Nella, Clara Bohm, Claudio Rigatto, Paul Komenda, Sue Boreskie, Carrie Solmundson, Leanne Kosowan, Navdeep Tangri

**Affiliations:** 1 Department of Community Health Sciences, Max Rady Faculty of Health Sciences, University of Manitoba, Winnipeg, Canada; 2 Chronic Disease Innovation Centre, Seven Oaks General Hospital, Winnipeg, Canada; 3 Department of Community Health Sciences, Max Rady Faculty of Health Sciences, Manitoba Centre for Health Policy, University of Manitoba, Winnipeg, Canada; 4 Department of Family Medicine, Rady Faculty of Health Sciences, University of Manitoba, Manitoba, Canada; 5 Department of Internal Medicine, Section of Nephrology, Max Rady Faculty of Health Sciences, University of Manitoba, Winnipeg, Canada; 6 Reh-Fit Centre, Winnipeg, Canada; 7 Wellness Institute, Winnipeg, Canada

**Keywords:** Medical fitness facility, Health care utilization, Mortality, Mace

## Abstract

**Background:**

Physical inactivity is common among older adults and is associated with poor health outcomes. Medical fitness facilities provide a medically focused approach to physical fitness and can improve physical activity in their communities. This study aimed to assess the relationship between membership in the medical fitness model and all-cause mortality, health care utilization, and major adverse cardiac events in older adults.

**Methods:**

A propensity weighted retrospective cohort study linked individuals that attended medical fitness facilities to provincial health administrative databases. Older adults who had at least 1 year of health coverage from their index date between January 1st, 2005 to December 31st 2015 were included. Controls were assigned a pseudo-index date at random based on the frequency distribution of index dates in members. Members were stratified into low frequency attenders (< 1 Weekly Visits) and regular frequency attenders (> 1 Weekly Visits). Time to event models estimated the hazard ratios (HRs) for risk of all-cause mortality and major adverse cardiac event. Negative binomial models estimated the risk ratios (RRs) for risk of hospitalizations, outpatient primary care visits and emergency department visits.

**Results:**

Among 3,029 older adult members and 91,734 controls, members had a 45% lower risk of all-cause mortality (HR: 0.55, 95% CI: 0.50 – 0.61), 20% lower risk of hospitalizations (RR: 0.80, 95% CI: 0.75 – 0.84), and a 27% (HR: 0.72, 95% CI: 0.66 – 0.77), lower risk of a major adverse cardiovascular event. A dose–response effect with larger risk reductions was associated with more frequent attendance as regular frequency attenders were 4% more likely to visit a general practitioner for a routine healthcare visit (RR: 1.04, 95% CI: 1.01 – 1.07), but 23% less likely to visit the emergency department (RR: 0.87, 95% CI: 0.82 – 0.92).

**Conclusions:**

Membership at a medical fitness facility was associated with a decreased risk of mortality, health care utilization and cardiovascular events. The medical fitness model may be an alternative approach for public health strategies to promote positive health behaviors in older adult populations.

**Supplementary Information:**

The online version contains supplementary material available at 10.1186/s12877-024-05208-6.

## Introduction

A demographic shift is occurring as a higher proportion of individuals worldwide are entering older adulthood. In the US, it is projected that by 2030 1 in every 5 Americans will be over the age of 65 [1]. Furthermore, aging is associated with an increased prevalence of chronic disease, impairment and disability that is associated with functional decline. Studies have estimated that 70–80% of older adults will have at least one chronic condition in their lifetime [2, 3]. Therefore, it is important that as older adults progress through life that they maintain physical function, independence, quality of life, and have access to interventions that will prevent or delay the onset of illness or disability.

It is well established that engaging in regular physical activity (PA) can help prevent premature death, disability, morbidity, frailty and the occurrence of major non-communicable diseases [4–11]. However, even though the benefits of PA are well known, a low proportion of older adults are achieving the recommended PA levels outlined by international and national guidelines. In the US, the proportion of older adults meeting the recommended guidelines for PA range from 27–44% which decreases further in adults over the age of 80 [12, 13]. These levels of physical inactivity are associated with an annual economic burden of approximately $27 billion US dollars [14]. Interventions that engage older adults to increase their PA levels and promote other positive health behaviors are needed.

Medical fitness facilities (MFF) aim to attract a broad spectrum of individuals, including both in good health and populations with health risks, notably older adults and individuals managing chronic disease [15]. The central tenet of the medical fitness model is the provision of evidence-based, medically integrated programming to advocate lifestyle medicine. As compared to conventional fitness centers, the medical fitness model incorporates medical oversight and heightened supervision, and guidance. This involves a greater degree of staff education and training, integrating with health systems, facilitating the transition between acute hospital care and long-term medical services, and robust emergency response and safety protocols [15].

Membership at these facilities provides access to various forms of PA, including aerobic and resistance training equipment, indoor recreation amenities and an array of group fitness classes. Consequently, attendance at these facilities may be used as a surrogate marker for participation in PA. Additionally, they provide comprehensive health assessments, individualized wellness plans and health education initiatives. Coaching services encompass a spectrum of lifestyle factors, including nutrition, stress management, sleep hygiene, smoking cessation, and disease management.

The primary aim of this study was to determine the association between frequency of attendance at an MFF with all-cause mortality, risk of health care utilization (hospitalizations for any cause, emergency department visit and visit to a general practitioner) and major adverse cardiovascular events (MACE) in older adult members.

## Methods

We conducted a retrospective cohort study with an intention-to-treat analysis. We compared older adult members who attended either of two MFF in Winnipeg, Canada to general population controls. Controls were identified through linked provincial health registries, which capture all individuals obtaining health services in Canada’s single-payer universal health system.

### Data sources

Data was sourced from the Population Research Data Repository housed at the Manitoba Centre for Health Policy (MCHP) [16, 17]. Further details are displayed in Supplement 1. Repository data are de-identified, meaning sensitive information that could identify the individual is removed prior to inclusion in the repository. However, individuals’ data is linkable across databases using a scrambled coded identifier derived from an individuals’ 9-digit personal health identification number (PHIN).

The MFFs collect identifiers, including PHIN, first and last name, and date of birth. These databases were linked to the repository by Manitoba Health using PHIN if available, or identifying characteristics such as date of birth, sex and postal code. Both MFFs have introduced scanning systems in order to gain access to the facility.

### Study population

The intervention group included members (≥ 65 years) at the MFF who were living in the city of Winnipeg, Canada. These facilities are open to any member of the public to join. Members were included from the introduction of the facility scanning systems (January 1st, 2005 for the Wellness Institute and August 1st, 2006 at the Reh-Fit Centre) to December 31st, 2015. The intervention group was assigned an index date that matched their membership start date. Controls included adult residents of Winnipeg that were registered with the provincial health insurance registry under a single-payer health system between January 1st, 2005 to December 31st, 2015. A pseudo-index date was assigned to the control group based on the time difference between start and end dates in the intervention group. The frequency distributions of time differences were then applied at random to controls [18]. The control group was restricted to individuals who had a pseudo-index date before health registry end date, which would have indicated loss to follow up or death. Individuals who had index dates that were not between their health coverage dates, those who had < 1 year of health coverage prior to the index date, duplicate entries in the health registry, and those without a postal code which was used to assign socioeconomic status were excluded from the analysis.

### Data collection

Demographic data was collected by linking scrambled 9-digit PHIN to health registry databases. Co-morbidities were assessed using validated co-morbidity indexes using well-defined *ICD-9-CM* and *ICD-10-CA* codes collected from physician and hospital claims [19, 20]. Further details are displayed in Supplement 2. Income quintiles were used as a proxy for socioeconomic status by linking postal codes to dissemination areas that are comprised of an average population of 400–700 persons providing data on average household income based on national census data [21].

### Exposures

The intervention group included new registered older adult members (≥ 65 years). Data was captured from each respective MFF on when members scanned in to access the facility to assess our dose response relationship. Members were stratified into two groups based on the total number of visits over the total duration in weeks of their membership during the study period: low frequency attenders (< 1 visits per week) and regular frequency attenders (> 1 visits per week).

### Outcomes

The primary outcome was time to all-cause mortality, with date of death ascertained from the health insurance registry data. Individuals were censored at the end of the study period or loss to follow-up. Individuals were considered lost to follow-up if they moved away from the province, or had their health coverage terminated for unknown reasons.

Secondary outcomes for health care utilization were assessed as the frequency of total visits from start of index or pseudo-index date to date of death or end of study period. CIHI-DAD database was used to determine inpatient hospitalization visits. A visit to the hospital was defined as a single stay (> 24 h), irrespective of a possible transfer to a different hospital. Visits to a family physician/general practitioner or nurse practitioner were ascertained from the Medical Claims databases. This definition has been validated in other population based studies [22]. Emergency department visits were determined from the ADT and EDIS databases. A visit to the emergency department was determined as a single date visit, irrespective of possible transfer or visit to a different emergency department.

MACE events were identified by using the CIHI-DAD database. A MACE event was the first incident of a heart failure, myocardial infarction, stroke, or cardiovascular related death.[23–28]. Further details are displayed in Supplement 3. MACE was ascertained starting from the index date in the intervention groups, and pseudo-index date in the controls until the end of the study period, death, or loss to follow-up.

### Statistical analysis

Characteristics were presented by membership status at MFF, with categorical variables presented as frequencies and percentages, and continuous variables as means and SD. A predicted probability (propensity score) of being assigned to the intervention group was developed using a logistic regression model that incorporated age, sex, income quintile, index year, and co-morbidities. A multinomial logistic regression model that incorporated the same covariates was used to determine propensity scores for the dose–response relationship [29]. Propensity scores were then used to estimate the treatment effect by the inverse probability treatment weighting (IPTW) adjustment method [30, 31]. In order to account for extreme weights, stabilized weights were used [32, 33]. Balance in covariates between groups was assessed using the standardized mean difference (SMD) before and after IPTW, with a balanced covariate having a SMD < 0.1 after IPTW [34, 35].

The association of the intervention with the outcome of time to all-cause mortality was analyzed with Cox proportional hazards regression models. Schoenfeld residuals were plotted against rank failure times to determine violation of the proportional hazards assumption by visual inspection. Negative binomial regression models were used to analyze the association between membership and the rate of hospitalization. Similar models were applied in the dose response cohort. Statistical analyses were performed using SAS/STAT® software, version 9.4 (Cary NC, USA).

## Results

### Patient characteristics

**Fig. 1:**
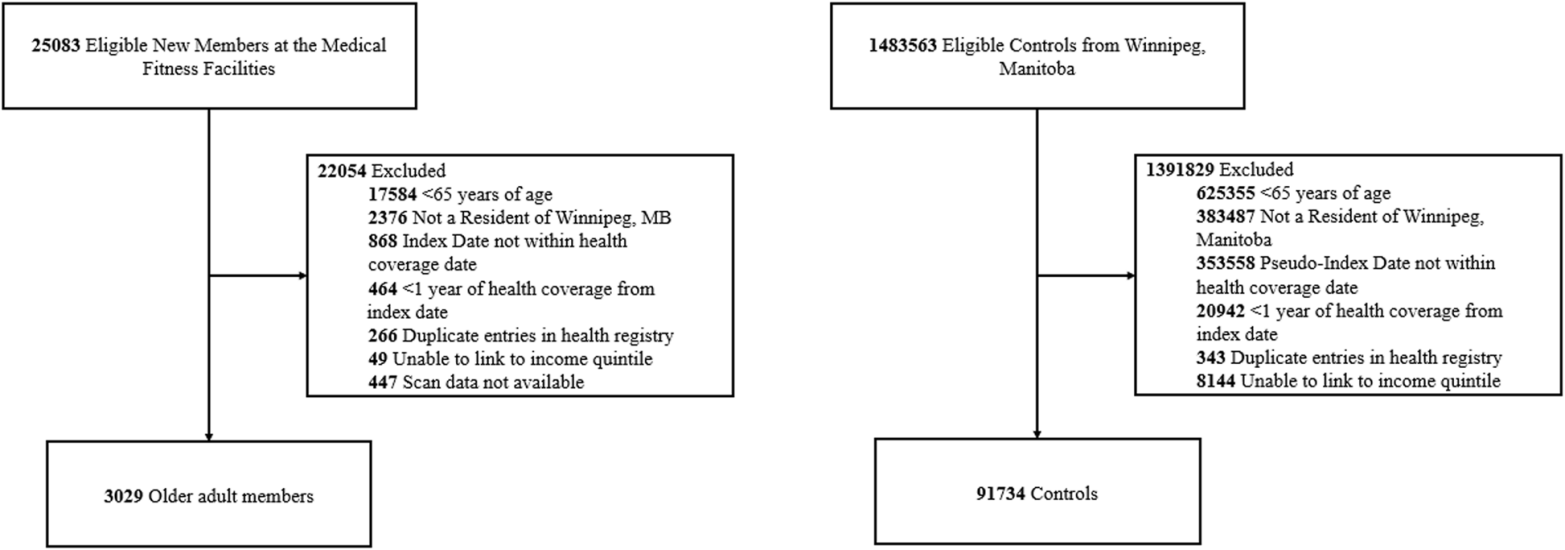
Strobe Diagram

A total of 91,734 members at an MFF were included in the intervention group and 3029 in the control group (Fig. 1). Among the intervention group 1754 members were low frequency attenders, 1275 were regular frequency attenders. The median (IQR) number of weekly visits in the overall member population was 0.84 (0.39, 1.45) and 0.44 (0.23, 0.71), 1.61 (1.24, 2.18) in low frequency attenders and regular frequency attenders, respectfully.

Older adult members had a higher prevalence of myocardial infarction, were more likely to be from a higher income quintile and had a lower prevalence of coronary artery disease and dementia at baseline as compared to controls (Table 1). Low frequency attenders and regular frequency attenders had a higher prevalence of previously diagnosed cancer, depression and myocardial infarction, a lower prevalence of dementia and were more likely to be from a higher income quintile, as compared to controls (Table 1). All covariates were balanced with a SMD less than 0. Further details are displayed in Supplement 4.

**Table 1:** Baseline characteristics before and after stabilized IPTW

	Unweighted				Stabilized IPTW^a^				
	Controls	Members	≤ 1 Weekly	> 1 Weekly	Controls	Members	Controls	≤ 1 Weekly	> 1 Weekly
**N**	91,734	3029	1754	1275	91,731.2	3019.6	91,734.7	1756.6	1258.5
Covariates									
Age (65 – 70 yrs)	26,153 (28.5)	1416 (46.8)	804 (45.8)	612 (48.0)	38,917.4 (42.4)	1387.7 (46.0)	26,688.8 (29.1)	508.0 (28.9)	377.4 (30.0)
Age (70 – 75 yrs)	20,270 (22.1)	771 (25.5)	458 (26.1)	313 (24.6)	26,687.7 (29.1)	884.8 (29.3)	20,368.3 (22.2)	390.1 (22.2)	293.9 (23.4)
Age (75 + yrs)	45,311 (49.4)	842 (27.8)	492 (28.1)	350 (27.5)	20,367.6 (22.2)	686.6 (22.7)	44,677.6 (48.7)	858.6 (48.9)	587.2 (46.7)
Male Sex, n (%)	38,770 (42.3)	1431 (47.2)	796 (45.4)	635 (49.8)	44,675.9 (48.7)	1448.07 (48.0)	52,815.8 (57.6)	953.0 (54.3)	665.5 (52.9)
Previous diagnosis of, n (%)									
Myocardial Infarction	7072 (7.7)	330 (10.9)	175 (10.0)	155 (12.1)	7165.3 (7.8)	232.1 (7.7)	7165.6 (7.8)	144.5 (8.2)	93.2 (7.4)
Congestive Heart Failure	12,230 (13.3)	302 (10.0)	188 (10.7)	114 (8.9)	12,130.9 (13.2)	376.7 (12.5)	12,131.4 (13.2)	236.2 (13.4)	135.7 (10.8)
Peripheral Vascular Disease	10,117 (11.0)	320 (10.6)	182 (10.4)	138 (10.8)	10,104 (11.0)	357.5 (11.8)	10,104.4 (11.0)	212.0 (12.1)	147.5 (11.7)
Cerebrovascular Disease	14,494 (15.8)	427 (14.1)	275 (15.7)	152 (11.9)	14,442.8 (15.7)	456.4 (15.1)	14,443.3 (15.7)	269.9 (15.4)	181.8 (14.4)
Dementia	7537 (8.2)	99 (3.3)	60 (3.4)	39 (3.1)	7391.5 (8.1)	227.4 (7.5)	7391.8 (8.1)	130.2 (7.4)	103.2 (8.2)
COPD	16,839 (18.4)	511 (16.9)	324 (18.5)	187 (14.7)	16,793.8 (18.3)	537.2 (17.8)	16,794.3 (18.3)	314.4 (17.9)	219.5 (17.4)
Rheumatic Disease	5838 (6.4)	174 (5.7)	104 (5.9)	70 (5.5)	5820.0 (6.3)	213.5 (7.1)	5820.3 (6.3)	121.0 (6.9)	94.6 (7.5)
Peptic Ulcer Disease	4673 (5.1)	134 (4.4)	84 (4.8)	50 (3.9)	15,069.2 (2.9)	538.8 (2.8)	4653.7 (5.1)	95.6 (5.4)	64.1 (5.1)
Cirrhosis	4079 (4.5)	141 (4.7)	81 (4.6)	60 (4.7)	21,539.4 (4.2)	818.5 (4.2)	4085.2 (4.5)	83.8 (4.8)	61.3 (4.9)
Diabetes	24,280 (26.5)	769 (25.4)	458 (26.1)	311 (24.4)	24,247.4 (26.4)	805.2 (26.7)	24,248.3 (26.4)	471.5 (26.8)	331.3 (26.3)
Paraplegia and Hemiplegia	2378 (2.6)	67 (2.2)	46 (2.6)	21 (1.7)	2366.8 (2.6)	72.8 (2.4)	2366.9 (2.6)	37.8 (2.2)	34.4 (2.7)
Renal Disease	5178 (5.6)	123 (4.1)	76 (4.3)	47 (3.7)	5131.3 (5.6)	177.1 (5.9)	5131.5 (5.6)	109.3 (6.2)	66.3 (5.4)
Cancer	21,302 (23.2)	799 (26.4)	476 (27.1)	323 (25.3)	21,394.5 (23.3)	699.2 (23.2)	21,395.4 (23.3)	418.0 (23.8)	281.8 (22.4)
Metastatic Carcinoma	2137 (2.3)	63 (2.1)	34 (1.9)	29 (2.3)	2129.5 (2.3)	61.6 (2.0)	2129.6 (2.3)	37.3 (2.1)	22.9 (1.8)
STI	41 (0.04)	< 6 (0.1)	< 6 (0.1)	< 6 (0.1)	41.7 (0.1)	< 6 (0.1)	41.7 (0.1)	< 6 (0.1)	< 6 (0.1)
Anxiety Disorder	699 (0.8)	23 (0.8)	12 (0.7)	11 (0.9)	698.8 (0.8)	21.1 (0.7)	698.8 (0.8)	9.5 (0.5)	10.9 (0.9)
Depression	20,465 (22.3)	774 (25.6)	489 (27.9)	285 (22.4)	20,559.4 (22.4)	666.7 (22.1)	20,560.1 (22.4)	380.5 (21.7)	290.5 (23.1)
Hypertension	67,461 (73.5)	2204 (72.8)	1280 (73.0)	924 (72.5)	67,437.1 (73.5)	2223.5 (73.6)	67,439.8 (73.5)	1300.0 (74.0)	916.6 (72.8)
Coronary Artery Disease	2087 (68.9)	942 (31.1)	564 (32.2)	378 (29.7)	26,285.8 (28.7)	824.0 (27.3)	26,286.8 (28.7)	482.1 (27.5)	335.2 (26.6)
Index Year, n (%)									
Index Year 2005	6318 (6.9)	171 (5.7)	91 (5.2)	80 (6.3)	6281.3 (6.9)	202.8 (6.7)	6281.5 (6.9)	122.8 (7.0)	83.2 (6.6)
Index Year 2006	8539 (9.3)	298 (9.8)	198 (11.3)	100 (7.8)	8553.5 (9.3)	259.3 (8.6)	8553.9 (9.3)	145.4 (8.3)	110.3 (8.8)
Index Year 2007	9347 (10.2)	266 (8.8)	172 (9.8)	94 (7.4)	9304.8 (10.1)	304.6 (10.0)	9305.2 (10.1)	170.7 (9.7)	134.3 (10.7)
Index Year 2008	8193 (8.9)	234 (7.7)	148 (8.4)	86 (6.8)	8157.8 (8.9)	277.2 (9.2)	8158.1 (8.9)	161.4 (9.2)	114.4 (9.1)
Index Year 2009	9516 (10.4)	291 (9.6)	180 (10.3)	111 (8.7)	9491.9 (10.4)	285.5 (9.5)	9492.2 (10.4)	173.2 (9.9)	110.0 (8.7)
Index Year 2010	8341 (9.1)	235 (7.8)	156 (8.9)	79 (6.2)	8301.9 (9.1)	274.3 (9.1)	8302.2 (9.1)	156.2 (8.9)	123.6 (9.8)
Index Year 2011	7879 (8.6)	244 (8.1)	135 (7.7)	109 (8.6)	7863.6 (8.6)	262.9 (8.7)	7864.0 (8.6)	156.2 (8.9)	123.6 (9.8)
Index Year 2012	8351 (9.1)	338 (11.2)	208 (11.9)	130 (10.2)	8411.5 (9.2)	283.3 (9.4)	8411.8 (9.2)	171.0 (9.7)	110.9 (8.8)
Index Year 2013	8954 (9.8)	385 (12.7)	213 (12.1)	172 (13.5)	9040.3 (9.9)	307.6 (10.2)	9040.6 (9.9)	181.6 (10.3)	124.5 (9.9)
Index Year 2014	8501 (9.3)	299 (9.9)	164 (9.4)	135 (10.6)	8518.9 (9.3)	290.2 (9.6)	8519.2 (9.3)	174.2 (9.9)	115.8 (9.2)
Index Year 2015	7795 (8.5)	268 (8.9)	89 (5.1)	179 (14.0)	7805.7 (8.5)	271.8 (9.0)	7806.1 (8.5)	144.2 (8.2)	124.0 (9.9)
Income Quintiles, n (%)									
1 (Low)	20,508 (22.4)	364 (12.0)	224 (12.8)	140 (11.0)	20,204.1 (22.0)	667.6 (22.1)	20,204.8 (22.0)	378.1 (21.5)	288.7 (22.9)
2	18,651 (20.3)	510 (16.8)	284 (16.2)	226 (17.7)	18,547.6 (20.2)	605.8 (20.1)	18,548.3 (20.2)	361.8 (20.6)	257.7 (20.5)
3	18,522 (20.2)	613 (20.2)	339 (19.3)	274 (21.5)	18,522.1 (20.2)	600.0 (19.9)	18,522.8 (20.2)	347.2 (19.8)	242.2 (19.2)
4	17,263 (18.8)	681 (22.5)	384 (21.9)	297 (23.3)	17,370.8 (18.9)	575.02 (19.0)	17,371.5 (18.9)	338.8 (19.3)	227.8 (18.1)
5 (High)	16,790 (18.3)	861 (28.4)	523 (29.8)	338 (26.5)	17,086.6 (18.6)	571.1 (18.9)	17,087.3 (18.6)	330.8 (18.8)	242.1 (19.2)

*Abbreviations*: I*PTW* Inverse probability treatment weighting *COPD*, Chronic Obstructive Pulmonary Disease.

^a^Standardized mean difference was < 0.1 in all group comparisons

### Propensity scores and IPTW analyses

**Fig. 2:**
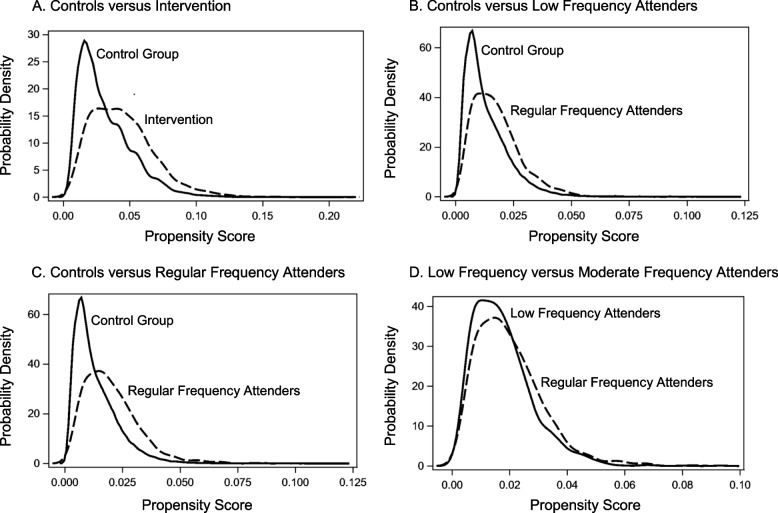
Comparison of propensity scores between study groups

Propensity scores demonstrated significant overlap between controls and all study cohorts, satisfying the positivity assumption of propensity score methods (Fig. 2A-D). The mean (SD) stabilized weight in controls was 0.99 (0.02) and intervention group was 0.99 (0.70). Low frequency attenders, regular frequency attenders and controls had mean (SD) stabilized weights of 1.00 (0.74), 0.99 (0.77), and 1.00 (0.02), respectfully.

### All-cause mortality

The median follow-up time was 2752 and 3016 days in the control and intervention group, respectively. The total number of deaths were 20,427 (22.3%) and 297 (9.8%) in the control and intervention group, respectfully. Compared to controls, the intervention group demonstrated a lower hazards risk of all-cause mortality in unweighted and stabilized IPTW models (HR = 0.40, 95% CI 0.36—0.45; HR = 0.55, 95% CI 0.50 – 0.61) (Table 2). In a subgroup analysis based on sex, females were associated with a lower hazards risk of all-cause mortality in stabilized IPTW models (HR = 0.43, 95% CI 0.37 – 0.50) as compared to males (HR = 0.65, 95% CI 0.57 – 0.74) (Supplement 5). No differences were seen based on age (Supplement 6).

**Table 2:** Cox proportional hazards models of time to all-cause mortality and MACE +

	**All-Cause Mortality**		**Major Adverse Cardiovascular Event**^**a**^	
**Model (Ref = Controls)**	**HR**	**95% CI**	**HR**	**95% CI**
Main				
Unadjusted	0.40	0.36 – 0.49	0.58	0.54 – 0.63
Stabilized IPTW	0.55	0.50 – 0.61	0.72	0.66 – 0.77
Dose Response				
Unadjusted				
Low Frequency Attenders	0.41	0.36 – 0.48	0.76	0.75 – 0.78
Regular Frequency Attenders	0.38	0.32 – 0.45	0.60	0.59 – 0.62
Stabilized IPTW				
Low Frequency Attenders	0.55	0.48 – 0.62	0.77	0.70 – 0.85
Regular Frequency Attenders	0.54	0.47 – 0.63	0.61	0.54 – 0.69

*Abbreviations* *Ref* Reference, *HR* Hazards Ratio, *CI* Confidence Interval, *IPTW* Inverse probability treatment weighting

^a^MACE + is defined as a hospitalization (> 24 h) for MI, Stroke or HF OR Cardiovascular Death

The median follow-up time was 3153 and 2895 and the total number of deaths were 181 (10.3%) and 116 (9.1%) in the low frequency attenders and regular frequency attenders, respectfully. All groups were associated with a lower hazards risk of all-cause mortality in stabilized IPTW models (low frequency attenders: HR = 0.55, 95% CI 0.48—0.62; regular frequency attenders: HR = 0.54, 95% CI 0.47—0.63), however a significant dose response relationship was not observed (Table 2). In a subgroup analysis based on sex, females who attended regularly were associated with a lower hazards risk of all-cause mortality (HR = 0.37, 95% CI 0.29 – 0.49) as compared to males (HR = 0.68, 95% CI 0.56 – 0.82) in stabilized IPTW models (Supplement 5). No differences were seen based on age (Supplement 6).

### Rate of hospitalizations

Compared to controls, older members at MFFs had a lower risk of hospitalization in the stabilized IPTW model (RR = 0.80, 95% CI 0.75—0.84) (Table 3). A dose response effect was evident as increased attendance had a lower risk of hospitalization (low frequency attenders: RR = 0.85, 95% CI 0.79—0.91; regular frequency attenders: RR = 0.72, 95% CI 0.66—0.79) (Table 3). Females who attended regularly had a lower risk of hospitalizations in stabilized IPTW models (RR = 0.57, 95% CI 0.50 – 0.65) as compared to males (RR = 0.85, 95% CI 0.76 – 0.94) (Supplement 5). No differences were seen based on age (Supplement 6).

**Table 3:** Negative binomial regression models

	**Hospitalizations**		**ED visits**		**Physician visits**	
Model (Ref = Controls)	RR	95% CI	RR	95% CI	RR	95% CI
Main						
Unadjusted	0.73	0.69 – 0.77	0.78	0.73 – 0.82	1.00	0.98 – 1.03
Stabilized IPTW	0.80	0.75 – 0.84	0.87	0.82 – 0.92	1.04	1.01 – 1.07
Dose Response						
Unadjusted						
Low Frequency Attenders	0.80	0.74 – 0.85	0.84	0.78 – 0.90	1.02	0.99 – 1.06
Regular Frequency Attenders	0.64	0.59 – 0.70	0.68	0.62 – 0.75	0.98	0.94 – 1.02
Stabilized IPTW						
Low Frequency Attenders	0.85	0.79 – 0.91	0.93	0.87 – 1.01	1.03	0.99 – 1.06
Regular Frequency Attenders	0.72	0.66 – 0.79	0.76	0.69 – 0.83	1.05	1.01 – 1.10

*Abbreviations*: *Ref* Reference, *RR* Rate Ratio, *CI* Confidence Interval, *ED* Emergency Department, *IPTW* inverse probability treatment weighting

### Rate of physician visits

Compared to controls, older members at MFFs had a higher risk of a physician visit in the stabilized IPTW model (RR = 1.04, 95% CI 1.01 – 1.07) (Table 3). A dose response effect was evident as regular frequency attenders had a higher risk of a physician visit (low frequency attenders: RR = 1.03, 95% CI 0.99 – 1.06; regular frequency attenders: RR = 1.05, 95% CI 1.01 – 1.10) (Table 3).

### Rate of emergency department visits

Compared to controls, members at MFF had a lower risk of an emergency department visit in the stabilized IPTW model (RR = 0.87, 95% CI 0.82 – 0.92) (Table 3). A dose response effect was apparent as regular frequency attenders had a lower risk of an emergency department visit (low frequency attenders: RR = 0.93, 95% CI 0.87 – 1.01; regular frequency attenders: RR = 0.76, 95% CI 0.69 – 0.83) (Table 3).

### Major adverse cardiovascular event

The median follow-up time was 2317 and 2626 days in the control and intervention group, respectively. The total number of MACE events were 26,716 (29.1%) and 582 (19.2%) in the control and intervention group, respectfully. Compared to controls, older members at MFF had a lower risk of a MACE event in the stabilized IPTW model (HR = 0.72, 95% CI 0.66 – 0.77) (Table 3).

The median follow-up time was 2698 and 2534 and the total number of MACE events were 378 (21.6%) and 204 (16.0%) in low frequency attenders and regular frequency attenders, respectfully. A dose response effect was apparent as regular frequency attenders had a lower risk of a MACE (low frequency attenders: HR = 0.77, 95% CI 0.70 – 0.85; regular frequency attenders: HR = 0.61, 95% CI 0.54 – 0.69) (Table 3).

## Discussion

Our findings suggest attendance at a medical fitness facility impacts longevity and health care utilization in older adults. Membership and attendance at MFFs were associated with improved survival, decreased risk of hospitalizations, emergency department visits, MACE and increased risk of a physician visit. Membership was associated with a 60% lower at risk of all-cause mortality, 20% lower at risk of a hospitalization, 13% lower at risk of an emergency department visit, 27% lower risk of a MACE and a 4% higher risk of visiting a physician over the course of the 6–8-year observation period. Risk for hospitalizations, emergency department visits and a MACE were even lower in those that attended more frequently. In addition, females had a lower risk of all-cause mortality, which was lower in those who attended more frequently, as compared to males. To our knowledge no other study has explored the effectiveness of the medical fitness model with long-term health outcomes in a community-dwelling older adult population.

A paucity of literature exists on the effectiveness of any exercise or health fitness interventions in generally healthy older adult populations, in respect to all-cause mortality and a MACE. In our study, we found a 60% reduced risk of all-cause mortality in older adult members and a 27% lower risk of a MACE, compared to generally healthy controls. In 19,000 community-dwelling, Australian, older adults over a follow-up period of 10 years, researchers found that physically active men and women (based on self-reported surveys) had a 20% and 40% reduction in mortality risk, respectively, after adjusting for chronic conditions, lifestyle factors and body mass index, as compared to sedentary individuals [36]. More recently, a prospective cohort study of 2500 Finnish, older adults with a follow up of 12 years found a 35% lower risk in all-cause mortality [4]. They also determined the risk of CVD mortality and an incident CVD event to be 30% and 40% lower in moderately active and very active groups, respectfully, based on self-reported baseline PA levels. However, even though these studies showed similar effect estimates, they did not define covariates or outcomes by linking to administrative databases. Secondly, they did not adjust for socioeconomic status, which has been independently associated with mortality [37]. Thirdly, the Australian cohort had a disproportionate ratio of females to males (2:3) and the Finnish cohort defined older adults as 65–74 years of age, therefore, minimizing the generalizability of the findings to diverse older adult populations.

A dose response effect was not evident in our study with respect to all-cause mortality. A few studies have described a reduced dose response effect between PA levels and mortality [38, 39]. However, these studies were sensitive to recall bias, therefore, overestimating the effect due to social desirability bias. A dose response effect may have been evident if we further categorized our dose response groups based on the frequency of weekly attendance. However, sample size and propensity balancing issues did not permit these analyses. Low frequency attenders had a 22% lower risk and regular frequency attenders had a 38% lower risk of a MACE. Previous studies have found a dose response effect with CVD mortality as older adults spent more time engaging in PA per week [40].

Older adult members at an MFF were 20% less likely to have a hospitalization, 13% less likely to have a visit at an ED and 4% more likely to visit a general practitioner. Previous studies have shown inconsistent findings between PA and health care utilization among older adults. Researchers found a 30–50% reduction in risk of a hospitalization when active older adults (> 65) were compared to inactive counterparts [41, 42]. These studies, however, used survey data that based inferences on 1 year recall of healthcare utilization. This may have overestimated the magnitude of the effect due to recall bias. Conversely, Bucher et al. explored the association of a 6-month exercise intervention program on healthcare utilization in older adults and found no significant difference in hospitalizations between the intervention group and controls [43]. In a prospective study, researchers found a 50% and 28% reduced likelihood of ED visits, in a subgroup of older adults aged 78 and 85, respectively [44]. In respect to physician visits, investigators have demonstrated that regular PA is associated with significantly lower outpatient health care costs in older adults [41, 45]. However, more recently investigators examined the association between PA and physician visits in 28,000 Swedish adults by linking to national health administrative databases to determine outcomes [46]. Once investigators adjusted for age, sex, education and income the association between higher PA levels and physician visits was not significant. Wang et al. examined the influence of PA on ED claims in retirees by adjusting for known risk factors [45]. They verified ED visits with insurance claims databases. Compared to the sedentary control group, moderately active and active older adults were 40% and 35% less likely to have an ED claim, providing evidence for a dose response effect.

Our findings are novel and could be indicative of new positive health behaviors attained by more frequent attendance at an MFF. These positive health choices are reinforced and encouraged by the uniqueness of the medical fitness model since there is a focus on providing educational seminars for health promotion, healthy eating and disease prevention. This increased health awareness may be an additional factor contributing to the survival benefit of older adults at an MFF. Future studies should explore cost-effectiveness models based on various attendance or fully subsidized memberships. Additionally, determining the different profiles of members in regards to the types of activities, duration and intensity they are engaging in and its impact on long-term health outcomes, will provide health care professionals evidence to prescribe PA based on individualized factors. Furthermore, the medical fitness model provides a unique opportunity for members to build social networks and personalized relationships. Studies have found that the social relations developed during exercise are related to increased satisfaction with life and reduced loneliness [47, 48]. Increases in social health may be an important contributor to long-term health outcomes of older adults [49]. Thus, although attendees participated in exercise while at the facilities, social interactions may have also contributed to health benefits we observed. Future studies should explore the association of social factors that may contribute to the effectiveness of the medical fitness model.

One of the main strengths of this study is the large sample size which allowed us to accurately estimate the effect size of the intervention. We were able to objectively measure the frequency of attendance over a sustained period of time, with an extensive follow up period of 10 years. Linking to provincial health administrative databases provided the unique opportunity to minimize selection bias in our intervention and control groups using inverse treatment probability weighting. The main limitation to our study was that we were not able to control for lifestyle factors such as smoking, alcohol, and dietary habits and other unidentified confounders. We were not able to adjust for baseline health status or behavior, since individuals attending MFF’s may already have positive health seeking behaviors, causing concern for residual confounding. In addition, the available data at the Population Research Repository does not include baseline health status, BMI, frailty, individual level education status, proximity to MFF, social and personality factors that may have influenced the outcomes. Moreover, as we did not have data on PA intensity and duration, the dose–response relationship can only be based on the frequency of weekly attendance. In addition, our findings may not be generalizable to other traditional fitness centers and healthcare systems. Furthermore, although we adjusted for baseline comorbidities, changes in health status over the follow-up period may have influenced the effects of the medical fitness model. Lastly, given the known issues with long-term adherence to exercise, the majority of the attendance may have occurred at the beginning of their memberships, therefore, attendance patterns could not be controlled for as a time-varying covariate.

## Conclusion

Attendance at a medical fitness facility, which offers a multimodal approach aimed at lifestyle modification, was associated with improved long-term health outcomes in older adults regardless of frequency of attendance. The medical fitness model may be an alternative approach for public health efforts aimed at reducing physical inactivity and sedentary time in older adults.

### Supplementary Information

Supplementary Material 1.

## Data Availability

Data are available from the Manitoba Centre for Health Policy, University of Manitoba, for researchers who meet the criteria for access to confidential data. All data used in our analysis are publicly available and held by the Government of Manitoba or contracted agencies. Access to this data requires approval by the University of Manitoba Health Research Ethics Board and the Government of Manitoba’s Health Privacy Information Committee. Legal restrictions prevent the use of these data without approval form each of the individual trustees.
