# Supplementary material for: Impact of the medical fitness model on long term health outcomes in older adults
Source: BMC Geriatr. 2024 Aug 20;24:695. doi: 10.1186/s12877-024-05208-6 (PMC11337618; doi:10.1186/s12877-024-05208-6)
Supplement: Supplementary file 1 — Supplementary Material 1. [file 12877_2024_5208_MOESM1_ESM.docx]

**Supplement 1. Databases accessed from the Population Research Data Repository at the Manitoba Centre for Health Policy**

| **Database** | **Years** | **Data Fields/Variables** | **Rationale** |
| --- | --- | --- | --- |
| **Vital Statistics** | 2004 - 2018 | Date of Birth, Date of Death | Construction of survival curves, mortality statistics, determination of age |
| **CIHI-DAD Hospital Discharge Abstracts (Manitoba Health)** | 2001-2018 | Diagnoses, interventions, date of admission, date of discharge | Determination of comorbidities and hospitalizations |
| **Medical Services (Manitoba Health)** | 2001-2018 | Diagnoses, interventions, date of service | Determination of comorbidities and primary care-related resource use |
| **Manitoba Health Insurance Registry** | 2004-2018 | Dates of coverage, gender, postal code, | Linkage of scrambled PHINs Determination of patient coverage for censoring and postal code for socioeconomic status |
| **Census** | 2004-2018 | Postal code, income quintile | Determination of socioeconomic status |
| **EDIS (WRHA)** | 2001-2018 | Date of service | Emergency room attendance |
| **ADT (WRHA)** | 2004-2018 | Date of service | Emergency room attendance |
| **Wellness Institute Membership Database** | 2005-2015 | Scrambled PHIN, membership start/end dates, frequency of total visits per year | Determination of Wellness Institute Membership and frequency of attendance |
| **Reh-Fit Centre Membership Database** | 2006-2015 | Scrambled PHIN, membership start/end dates, frequency of total visits per year | Determination of Reh-Fit Centre Membership and frequency of attendance |

Abbreviations: CIHI-DAD, Canadian Institute of Health Information – Discharge Abstract Database; EDIS, Emergency Department Information System; ADT; Admission, Discharge, Transfer System

**Supplement 2. Definitions and ICD-9-CM and ICD-10-CA codes of covariates in propensity model^a,b^**

| **Covariate** | **Definition** |
| --- | --- |
| **Myocardial infarction** | - One or more hospitalizations with diagnosis of ICD-9-CM codes 410.x, 412.x or ICD-10-CA codes I21.x, I22.x, I25.2; OR - One or more physician visits with diagnosis of ICD-9-CM codes 410, 412 |
| **Congestive heart failure** | - One or more hospitalizations with diagnosis of ICD-9-CM codes 398.91, 402.01, 402.11, 402.91, 404.01, 404.03, 404.11, 404.13, 404.91, 404.93, 425.4, 425.5, 425.6, 425.7, 425.8, 425.9, 428.x or ICD-10-CA codes I43.x, I50.x, I09.9, I11.0, I13.0, I13.2, I25.5, I42.0, I42.5, I42.6, I42.7, I42.9, P29.0; OR - One or more physician visits with diagnosis of ICD-9-CM codes 428 |
| **Peripheral vascular disease** | - One or more hospitalizations with diagnosis of ICD-9-CM codes 093.0, 437.3, 440.x, 441.x, 443.x, 447.1, 557.1, 557.9, V43.4 or ICD-10-CA codes I70.x, I71.x, I73.1, I73.8, I73.9, I77.1, I79.0, I79.2, K55.1, K55.8, K55.9, Z95.8, Z95.9; OR - One or more physician visits with diagnosis of ICD-9-CM codes 440, 441, 443 |
| **Cerebrovascular disease** | - One or more hospitalizations with diagnosis of ICD-9-CM codes 362.34, 430.x-438.x or ICD-10-CA codes G45.x, G46.x, H34.0, I60.x-I69.x; OR - One or more physician visits with diagnosis of ICD-9-CM codes 431-438; OR |
| **Dementia** | - One or more hospitalizations with diagnosis of ICD-9-CM codes 290.x or ICD-10-CA codes F00.x-F03.x, F05.1, G30.x, G31.1; OR - One or more physician visits with diagnosis of ICD-9_CM codes 290 |
| **Chronic obstructive pulmonary disease** | - One or more hospitalizations with diagnosis of ICD-9-CM codes 491.x, 492.x, 496.x or ICD-10-CA codes J41.x-J44.x; OR - One or more physician visits with diagnosis of ICD-9-CM codes 491, 492, 496 |
| **Rheumatic disease** | - One or more hospitalizations with diagnosis of ICD-9-CM codes 446.5, 710.0, 710.1, 710.4, 710.8, 710.9, 711.2, 714.x, 719.3, 720.x, 725.x, 728.5, 728.89, 729.30 or ICD-10-CA codes M05.x, M06.x, M31.5, M32.x-M34.x, M35.1, M35.3, M36.0; OR - One or more physician visits with diagnosis of ICD-9-CM codes 446, 710, 714, 720, 725 |
| **Peptic ulcer disease** | - One or more hospitalizations with diagnosis of ICD-9-CM codes 531.x-534.x or ICD-10-CA codes K25.x-K28.x - One or more physician visits with diagnosis of CID-10-CA codes 531-534 |
| **Liver disease** | - One or more hospitalizations with diagnosis of ICD-9-CM codes 070.22, 070.23, 070.33, 070.44, 070.54, 0706.0, 070.9, 456.1, 456.2, 570.x, 571.x, 572.2, 572.3, 572.5, 572.6, 572.7, 572.8, 573.3, 573.4, 573.8, 573.9, V42.7 or ICD-10-CA codes B18.x, K70.0-K70.4, K70.9, K71.1, K71.3-K71.5, K71.7, K72.1, L72.9, K73.x, K74.x, K76.0, K76.2-K76.8, K76.9, Z94.4, I85.0, I85.9, I86.4, I98.2; OR - One or more physician visits with diagnosis of ICD-9-CM codes 070, 456, 570, 571, 572, 573 |
| **Diabetes with or without complications** | - One or more hospitalizations with diagnosis of ICD-9-CM codes 250.1-250.9 or ICD-CA-10 codes E10.0-E10.9, E11.0-E11.9, E12.0-E12.9, E13.0-E13.9, E14.0-E14.9; OR - One or more physician visits with diagnosis of ICD-9-CM 250 |
| **Hemiplegia or paraplegia** | - One or more hospitalizations with diagnosis of ICD-9-CM codes ICD-9: 344.1, 342.x or ICD-CA_10 codes ICD-10: G04.1, G11.4, G80.1, G80.2, G81.x, G82.x, G83.0-G83.4, G83.9; OR - One or more physician visits with diagnosis of ICD-9-CM 342, 343, 344 |
| **Renal Disease** | - One or more hospitalizations with diagnosis of ICD-9-CM codes ICD-9: 403.01, 403.11, 403.91, 404.02, 404.12, 404.13, 404.92, 582.x, 583-583.7, 585.x, 586.x, 588.0, V42.0, V45.1, V56.x or ICD-10-CA codes I12.0, I13.1, N03.2-N03.7, N05.2-N05.7, N18.x, N19.x, N25.0, Z49.0-Z49.2, Z94.0, Z99.2; OR - One or more physician visits with diagnosis of ICD-9-CM codes 582, 583, 585, 586, 588, V56 |
| **Cancer** | - One or more hospitalizations with diagnosis of ICD-9-CM codes 140.x-172.x, 174.x-195.x, 200.x-208.x, 238.6 or ICD-10-CA codes C00.x-C26.x, C30.x-C34.x, C37.x-C41.x, C43.x, C45.x-C58.x, C60.x-C76.x, C81.x-C85.x, C88.x, C90.x-C97.x; OR - One or more physician visits with diagnosis of ICD-9-CM codes 140 – 172, 174 – 195, 200 – 208 |
| **Metastatic Carcinoma** | - One or more hospitalizations with diagnosis of ICD-9-CM codes 196.x – 199.x or ICD-10-CA codes C77.x-C80.x - One or more physician visits with diagnosis of ICD-9-CM codes 196-199 |
| **STI** | - One or more hospitalizations with diagnosis of ICD-9-CM codes 042.x-044.x or ICD-10-CA codes B20.x-B22.x, B24.x; OR - One or more physician visits with diagnosis of ICD-9-CM codes 042 – 044 |
| **Anxiety Disorder** | - One or more hospitalizations with diagnosis of ICD-9-CM codes 300.0, 300.2, 300.3, 300.7 or ICD-10-CA codes F40.x, F41.0, F41.1, F41.3, F41.8, F41.9, F42.0, F45.2; OR - One or more physician visits with diagnosis of ICD-9-CM codes 300 |
| **Depression** | - One or more hospitalizations with diagnosis of ICD-9-CM codes 296.2, 296.3, 296.5, 300.4, 309.x, 311.x or ICD-10-CA codes F20.4, F31.3-F31.5, F32.x, F33.x, F34.1, F41.2, F43.1, F43.2; OR - One or more physician visits with diagnosis of ICD-9-CM codes 296, 309, 311 |
| **Hypertension** | - One or more hospitalizations with diagnosis of ICD-9-CM codes 401.x-405.x or ICD-10-CA codes I10.x-I13.x, I15.x; O - One or more physician visits with diagnosis of ICD-9-CM codes 401 - 405 |
| **Coronary Artery Disease** | - One or more hospitalizations with diagnosis of ICD-9-CM codes 411.x, 413.x, 414.x or ICD-10-CA codes I20.x, I23.x, I24.x, I25.1, I25.3-I25.9; OR - One or more physician visits with diagnosis of ICD-9-CM codes 411, 413, 414 |
| **Age 65-70** | Age of cohort at index or pseudo-index date between the ages of 65 to <70 |
| **Ages 70 -75** | Age of cohort at index or pseudo-index date between the ages of 70 to <75 |
| **Ages 75+** | Ages of cohort at index or pseudo-index date over the age of 75 |
| **Sex** | Biological sex registered with Manitoba Health Insurance Registry |
| **Income Quintiles** | 20% population income quintile groups based on the average household income values and population count |
| **Index Year** | Year of member start date in intervention group and year of pseudo-index date in control group |

Abbreviations: ICD-9, International Classification of Diseases, Ninth Revision, Clinical Modification; ICD-10, International Classification of Diseases, Tenth Revision, Canadian Classification

^a^Definitions are based on the enhanced ICD-9-CM guidelines by Quan et al.

^b^Medical Services database only records ICD-9-CM codes at the 3 digit level.

**Supplement 3. Outcomes**

| **Outcomes** | **Definitions** |
| --- | --- |
| All-cause mortality | Death captured in the health registry if cancellation code = 2 |
| All-cause hospitalization | Total count of hospitalizations requiring a stay of at least 24 hours between index or pseudo-index date and study end date |
| Physician Visit | Total count of visits to a general practitioner or primary care nurse in an outpatient setting between index or pseudo-index date and study end date. Outpatient visits include office visits, walk-in clinics and home visits. Inpatient visits at a hospital or personal care home, outpatient surgeries and diagnostic tests and procedures are also not considered a physician visit |
| Emergency Department Visit | Total count of emergency department visits between index or pseudo-index date and study end date |
| Major Adverse Cardiovascular Event | An incident hospitalization event for an episode of: acute myocardial infarction, heart failure, stroke, unstable angina, angina pectoris, transient ischemic attack, ischemic heart disease, stroke and peripheral vascular disease  ICD-9: 402.01, 402.11, 402.91, 410-414, 420-432, 433.x1, 434.x1, 436, 440  ICD-10: G45, I11.0, I20.0, I20.9, I21-I22, I25.1, I25.2, I50, I60-64, I70-79, Z95, G45 |

**Supplement 4. Standardized mean differences before and after stabilized IPTW**

|  | Controls vs Members | | Controls vs ≤ 1 Weekly | | Controls vs >1 Weekly | | ≤ 1 Weekly vs >1 Weekly | |
| --- | --- | --- | --- | --- | --- | --- | --- | --- |
| Covariates | Before IPTW | After IPTW | Before IPTW | After IPTW | Before IPTW | After IPTW | Before IPTW | After IPTW |
| Age (65 – 70 yrs) | 0.38^a^ | <0.01 | 0.36^a^ | <0.01 | 0.41^a^ | 0.02 | 0.04 | 0.02 |
| Age (70 – 75 yrs) | 0.08 | 0.01 | 0.09 | <0.01 | 0.06 | 0.03 | 0.04 | 0.03 |
| Age (75+ yrs) | 0.45^a^ | 0.01 | 0.45^a^ | <0.01 | 0.46^a^ | 0.04 | 0.01 | 0.04 |
| Male Sex, n (%) | 0.10^a^ | 0.07 | 0.06 | 0.07 | 0.15^a^ | 0.09 | 0.09 | 0.03 |
| Myocardial Infarction | 0.11^a^ | <0.01 | 0.08 | 0.02 | 0.15^a^ | 0.02 | 0.07 | 0.03 |
| Congestive Heart Failure | 0.10^a^ | 0.02 | 0.08 | 0.01 | 0.14^a^ | 0.08 | 0.06 | 0.08 |
| Peripheral Vascular Disease | 0.01 | 0.03 | 0.02 | 0.03 | 0.01 | 0.02 | 0.01 | 0.01 |
| Cerebrovascular Disease | 0.05 | 0.02 | <0.01 | 0.01 | 0.11^a^ | 0.04 | 0.11^a^ | 0.03 |
| Dementia | 0.21^a^ | 0.02 | 0.21^a^ | 0.02 | 0.23^a^ | 0.01 | 0.02 | 0.03 |
| COPD | 0.04 | 0.01 | <0.01 | 0.01 | 0.10^a^ | 0.02 | 0.10^a^ | 0.01 |
| Rheumatic Disease | 0.03 | 0.03 | 0.02 | 0.02 | 0.04 | 0.05 | 0.02 | 0.02 |
| Peptic Ulcer Disease | 0.03 | 0.01 | 0.01 | 0.02 | 0.06 | <0.01 | 0.04 | 0.02 |
| Cirrhosis | 0.01 | 0.01 | 0.01 | 0.02 | 0.01 | 0.02 | <0.01 | <0.01 |
| Diabetes | 0.02 | 0.01 | 0.01 | 0.01 | 0.05 | <0.01 | 0.04 | 0.01 |
| Paraplegia and Hemiplegia | 0.02 | 0.01 | <0.01 | 0.03 | 0.07 | 0.01 | 0.07 | 0.04 |
| Renal Disease | 0.07 | 0.01 | 0.06 | 0.03 | 0.09 | 0.01 | 0.03 | 0.04 |
| Cancer | 0.07 | <0.01 | 0.09 | 0.01 | 0.05 | 0.02 | 0.04 | 0.03 |
| Metastatic Carcinoma | 0.02 | 0.02 | 0.03 | 0.01 | <0.01 | 0.04 | 0.02 | 0.02 |
| STI | 0.01 | <0.01 | 0.01 | <0.01 | 0.01 | <0.01 | 0.01 | <0.01 |
| Anxiety Disorder | <0.01 | 0.01 | 0.01 | 0.03 | 0.01 | 0.01 | 0.02 | 0.04 |
| Depression | 0.08 | 0.01 | 0.13^a^ | 0.02 | <0.01 | 0.02 | 0.13^a^ | 0.03 |
| Hypertension | 0.02 | <0.01 | 0.01 | 0.01 | 0.02 | 0.02 | 0.01 | 0.03 |
| Coronary Artery Disease | 0.06 | 0.03 | 0.08 | 0.03 | 0.02 | 0.05 | 0.05 | 0.02 |
| Index Year 2005 | 0.05 | 0.01 | 0.25^a^ | 0.01 | 0.31^a^ | 0.02 | 0.06 | 0.03 |
| Index Year 2006 | 0.02 | 0.03 | 0.11^a^ | 0.01 | 0.07 | 0.01 | 0.04 | <0.01 |
| Index Year 2007 | 0.05 | <0.01 | 0.02 | 0.01 | 0.03 | 0.02 | 0.05 | 0.01 |
| Index Year 2008 | 0.04 | 0.01 | 0.08 | 0.01 | 0.11^a^ | 0.02 | 0.03 | 0.03 |
| Index Year 2009 | 0.03 | 0.03 | 0.27^a^ | 0.01 | 0.20^a^ | 0.02 | 0.07 | 0.01 |
| Index Year 2010 | 0.05 | <0.01 | 0.07 | 0.01 | 0.02 | 0.01 | 0.05 | 0.01 |
| Index Year 2011 | 0.02 | <0.01 | 0.07 | 0.04 | 0.05 | 0.02 | 0.12^a^ | 0.02 |
| Index Year 2012 | 0.07 | 0.01 | 0.01 | 0.01 | 0.10^a^ | 0.02 | 0.09 | 0.03 |
| Index Year 2013 | 0.09 | 0.01 | 0.02 | 0.01 | 0.08 | 0.01 | 0.06 | <0.01 |
| Index Year 2014 | 0.02 | 0.01 | <0.01 | 0.02 | 0.06 | 0.05 | 0.05 | 0.04 |
| Index Year 2015 | 0.01 | 0.02 | 0.01 | 0.01 | 0.11^a^ | 0.03 | 0.10^a^ | 0.03 |
| Income Quintile 1 | 0.28^a^ | <0.01 | 0.03 | 0.01 | <0.01 | <0.01 | 0.03 | 0.01 |
| Income Quintile 2 | 0.09 | <0.01 | 0.09 | 0.02 | 0.04 | 0.01 | 0.05 | 0.03 |
| Income Quintile 3 | <0.01 | 0.01 | 0.08 | 0.02 | 0.12^a^ | <0.01 | 0.04 | 0.01 |
| Income Quintile 4 | 0.09 | <0.01 | <0.01 | 0.02 | 0.04 | <0.01 | 0.04 | 0.02 |
| Income Quintile 5 | 0.24^a^ | 0.01 | 0.14^a^ | 0.01 | 0.18^a^ | 0.05 | 0.31^a^ | 0.06 |

Abbreviations: IPTW, Inverse probability treatment weighting; COPD, Chronic Obstructive Pulmonary Disease; STI, sexually transmitted infection

**^a^**Standardized mean difference was >0.1

**Supplement 5. Cox proportional hazards models of time to all-cause mortality and negative binomial models of hospitalizations between males and females**

|  | All-Cause Mortality | | Hospitalizations | |
| --- | --- | --- | --- | --- |
| Model (Ref = Controls) | HR | 95% CI | RR | 95% CI |
| Sex = Females |  |  |  |  |
| Main |  |  |  |  |
| Unadjusted | 0.27 | 0.23 – 0.33 | 0.69 | 0.60 – 0.75 |
| Stabilized IPTW | 0.43 | 0.37 – 0.50 | 0.76 | 0.67 – 0.85 |
| Dose Response |  |  |  |  |
| Unadjusted |  |  |  |  |
| Low Frequency Attenders | 0.31 | 0.24 – 0.39 | 0.73 | 0.66 – 0.80 |
| Regular Frequency Attenders | 0.22 | 0.16 – 0.32 | 0.49 | 0.43 – 0.56 |
| Stabilized IPTW |  |  |  |  |
| Low Frequency Attenders | 0.45 | 0.37 – 0.56 | 0.80 | 0.72 – 0.88 |
| Regular Frequency Attenders | 0.37 | 0.29 – 0.49 | 0.57 | 0.50 – 0.65 |
| Sex = Males |  |  |  |  |
| Main |  |  |  |  |
| Unadjusted | 0.52 | 0.45 – 0.60 | 0.63 | 0.56 – 0.72 |
| Stabilized IPTW | 0.65 | 0.57 – 0.74 | 0.74 | 0.65 – 0.84 |
| Dose Response |  |  |  |  |
| Unadjusted |  |  |  |  |
| Low Frequency Attenders | 0.53 | 0.44 – 0.64 | 0.87 | 0.78 – 0.96 |
| Regular Frequency Attenders | 0.51 | 0.41 – 0.63 | 0.77 | 0.69 – 0.87 |
| Stabilized IPTW |  |  |  |  |
| Low Frequency Attenders | 0.61 | 0.51 – 0.73 | 0.90 | 0.80 – 0.99 |
| Regular Frequency Attenders | 0.68 | 0.56 – 0.82 | 0.85 | 0.76 – 0.94 |

Abbreviations: Ref, Reference; HR, Hazards Ratio, RR, Rate Ratio, CI, Confidence Interval; IPTW, inverse probability treatment weighting

**Supplement 6. Cox proportional hazards models of time to all-cause mortality and negative binomial models of hospitalizations between age groups**

|  | All-Cause Mortality | | Hospitalizations | |
| --- | --- | --- | --- | --- |
| Model (Ref = Controls) | HR | 95% CI | RR | 95% CI |
| Age (65-70 years) |  |  |  |  |
| Main |  |  |  |  |
| Unadjusted | 0.59 | 0.47 – 0.74 | 0.72 | 0.63 – 0.82 |
| Stabilized IPTW | 0.56 | 0.44 – 0.71 | 0.75 | 0.65 – 0.85 |
| Ages (70 – 75 years) |  |  |  |  |
| Main |  |  |  |  |
| Unadjusted | 0.73 | 0.58 – 0.91 | 0.70 | 0.65 – 0.85 |
| Adjusted | 0.76 | 0.61 – 0.94 | 0.75 | 0.64 – 0.89 |
| Ages (75+ years) |  |  |  |  |
| Main |  |  |  |  |
| Unadjusted | 0.43 | 0.36 – 0.51 | 0.74 | 0.64 – 0.86 |
| Adjusted | 0.50 | 0.43 – 0.59 | 0.80 | 0.68 – 0.93 |

Abbreviations: Ref, Reference; HR, Hazards Ratio, RR, Rate Ratio, CI, Confidence Interval; IPTW, inverse probability treatment weighting
